# 

**Published:** 1908-08-08

**Authors:** 


					" OFFICE]
T.i^phio Addr.??!. . ;-.y THE MODERN T,1"h??" Number
o.I.d.1., tdJik I r MEDICAL journal.
""EW SERIES. No. 74, VoJ.JII^ ^Ttttptiav
_ no.i4^.vdt.tliv. Saturday,
August 8tii, 1908.
\^i6elthreepence

				

